# Risk factors and management associated with postoperative cerebrospinal fluid leak after endoscopic endonasal surgery for pituitary adenoma

**DOI:** 10.3389/fsurg.2022.973834

**Published:** 2022-09-07

**Authors:** Bin Li, Sida Zhao, Qiuyue Fang, Ding Nie, Jianhua Cheng, Haibo Zhu, Chuzhong Li, Songbai Gui, Yazhuo Zhang, Peng Zhao

**Affiliations:** ^1^ Beijing Neurosurgical Institute, Capital Medical University, Beijing, China; ^2^ Department of Neurosurgery, Beijing Tiantan Hospital, Capital Medical University, Beijing, China

**Keywords:** cerebrospinal fluid leak, endoscopic endonasal surgery, pituitary adenoma, risk factor, management

## Abstract

**Objective:**

To determine risk factors and management for the development of a postoperative cerebrospinal fluid (CSF) leak after an endoscopic endonasal surgery (EES) for pituitary adenomas.

**Methods:**

The clinical data of 400 patients who underwent EES for resection of pituitary adenomas from December 2018 to November 2019 in the Department of Neurosurgery of Beijing Tiantan Hospital were retrospectively reviewed. Age, gender, body mass index (BMI), tumor size, Knosp grade, suprasellar extension grade, sellar floor erosion grade, repeated transsphenoidal surgery, intraoperative CSF leak, use of pedicled nasoseptal flap and lumbar drain were collected and analyzed.

**Results:**

Postoperative CSF leak occurred in 14 of 400 patients (3.5%). Age, gender, BMI, tumor size, Knosp grade and repeated transsphenoidal surgery were not risk factors for CSF leak. Suprasellar extension grade (≥B 6.0% vs. <B 1.4%; *p* = 0.024), sellar floor erosion grade (≥III 5.7% vs. <III 0.6%; *p* = 0.020) and intraoperative CSF leak (Yes 7.5% vs. No 2.0%; *p* = 0.009) were factors associated with an increased postoperative CSF leak rate.

**Conclusions:**

Higher suprasellar extension grade, higher sellar floor erosion grade and intraoperative CSF leak were risk factors for postoperative CSF leak after endoscopic treatment of pituitary adenoma. Strict skull base reconstruction including use of a pedicled nasoseptal flap and perioperative lumbar drainage may avoid postoperative CSF leak.

## Introduction

Pituitary adenoma (PA) is one of the common primary neoplasms of the central nervous system. It makes up approximately 10%–15% of all intracranial tumors (1, 2). Endoscopic endonasal surgery (EES) has become the best way to remove pituitary adenomas, with the development of neuroendoscopy equipment and technology. Postoperative cerebrospinal fluid (CSF) leak is the most important complication of EES. Postoperative CSF leak can increase the risk of intracranial infection, hospitalization time and costs (3). It is important to determine risk factors and management for the development of a postoperative CSF leak after the EES for resection of pituitary adenomas. According to previous studies, potential risk factors for CSF leak after EES include tumor size, body mass index (BMI), multiple EES and vascularized nasoseptal flap (4–6). However, there are many potential risk factors such as Knosp grade, suprasellar extension grade, sellar floor erosion grade and intraoperative CSF leak. These potential risk factors are rarely reported.

The management to prevent CSF leak after EES of pituitary adenomas is gradually improving. In 2006, the use of a vascular pedicled flap from the nasal septum mucoperiosteum was introduced, which has significantly optimized the skull base reconstruction technique (7). It reduces the incidence of CSF leak in the postoperative period after endonasal skull base surgery, because vascularized flaps promote faster and more complete healing by restoring the local blood (8). In addition, perioperative LD reduced the rate of postoperative CSF leak (6).

In our study, we comprehensively analyzed the risk factors of CSF leak after EES for pituitary tumor surgery. Based on these risk factors, we initially formulated a scheme to prevent CSF leak after EES for the resection of pituitary tumors. These risk factors include age, gender, BMI, tumor size, Knosp grade, suprasellar extension grade, sellar floor erosion grade, lumbar drain, repeated transsphenoidal surgery and intraoperative CSF leak.

## Materials and methods

### Study design

To determine risk factors and management for the development of a postoperative cerebrospinal fluid (CSF) leak after an endoscopic endonasal surgery (EES) for pituitary adenomas.

### Participants

Patients with pituitary adenoma who underwent EES between December 2018 and November 2019 in the Department of Neurosurgery of Beijing Tiantan Hospital affiliated to Capital Medical University were selected as the research subjects. All patients were treated by the same team, Neurosurgery Oncology 3 Ward. All patients' medical records and operative notes were reviewed in detail. All patients were followed up for at least 3 months.

### Variables

Accurately recorded the following information about the patients: age, gender, body mass index (BMI), tumor size, Knosp grade, suprasellar extension grade, sellar floor erosion grade, repeated transsphenoidal surgery, intraoperative CSF leak, use of pedicled nasoseptal flap and lumbar drain. Postoperative CSF leak was defined as a definite CSF leak within one month after EES. Laboratory tests indicated that fluid from the nose contained cerebrospinal fluid components (a definite CSF leak).

### Quantitative variables

**Figure 1:**
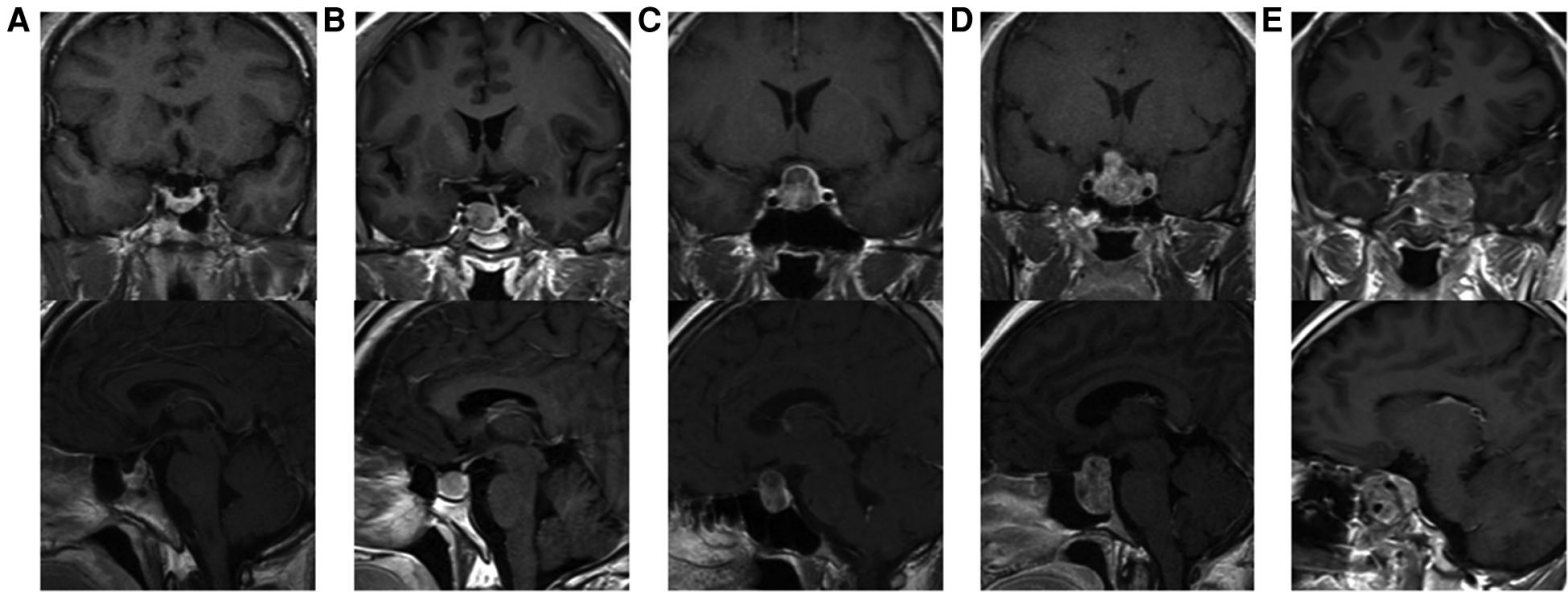
Knosp grades of patients. (**A**) Knosp 0, tumor is medial to medial tangent. (**B**) Knosp I, tumor extends to the space between the medial tangent and the intercarotid line. (**C**) Knosp II, tumor extends to the space between the intercarotid line and the lateral tangent. (**D**) Knosp III, tumor extends lateral to the lateral tangent. (**E**) Knosp IV, tumor with a complete encasement of intracavernous internal carotid artery.

**Figure 2:**
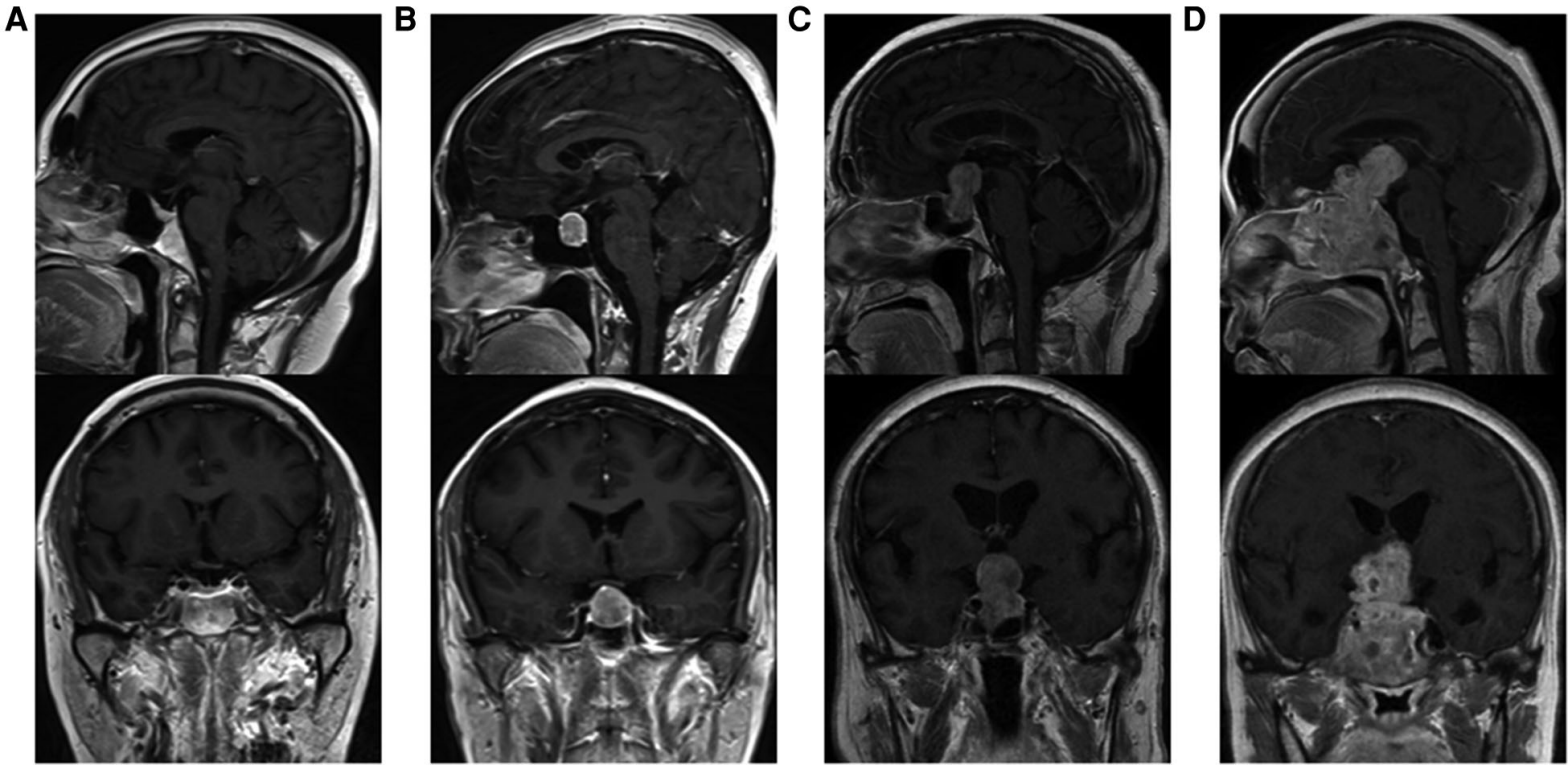
Suprasellar extension grades of patients. (**A**) Grade 0, no suprasellar extension. (**B**) Grade A, expanding into the suprasellar cistern. (**C**) Grade B, anterior recesses of the third ventricle obliterated. (**D**) Grade C, the floor of the third ventricle grossly displaced.

**Figure 3:**
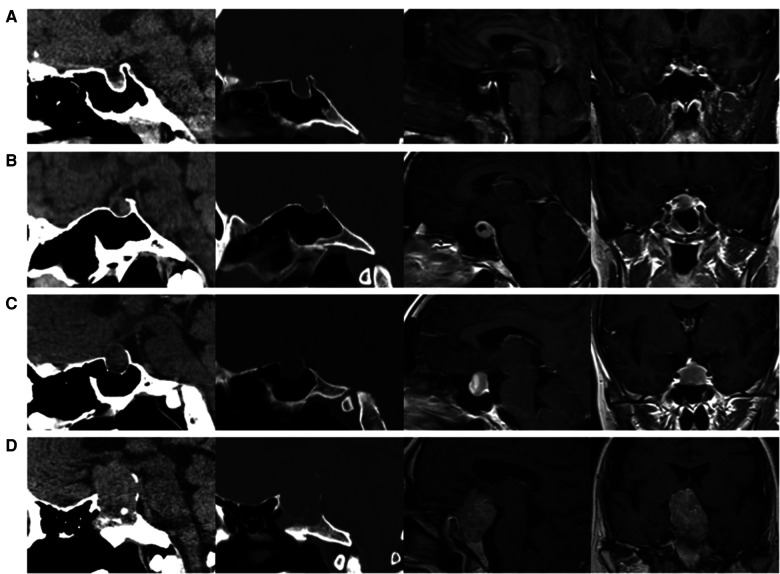
Sellar floor erosion grades of patients. (**A**) Grade I, sella normal or focally expanded; tumor <10 mm. (**B**) Grade II, sella enlarged; tumor ≥ 10 mm. (**C**) Grade III, localized sellar perforation. (**D**) Grade IV, diffuse destruction of the sellar floor.

Tumor size is represented by the longest distance of anteroposterior, transverse and vertical diameters. The determination of the Knosp grade of the cases is based on the 0-IV grade classification proposed by Professor Knosp (9) (Figure 1). Knosp grade can reflect parasellar extension of the tumor. The Hardy–Wilson classification (10) was used for the assessment of suprasellar extension grades and sellar floor erosion grades (Figures 2, 3). It is important to point out that since Hardy D and E grades reflect parasellar extension, we only used Hardy 0–C grades for the evaluation of suprasellar extension grade. The choice of a BMI of 24 kg/m^2^ as the cutoff for our analysis was based on the definition of overweight by the National Health, Family Planning Commission of the People's Republic of China. In this study, lumbar drain refers to the placement of a drainage tube before postoperative CSF leakage occurs. Lumbar drain is usually placed immediately after surgery or the day after surgery.

### Statistical analysis

To measurement variables, independent t-tests were used to compare the two groups of patients with and without CSF leaks. Chi-square tests were used to categorical variables. All independent variables that showed a significant correlation with dependent variables were placed in a multiple logistic forward stepwise regression. Multivariate logistic regression for predictors of postoperative CSF leak was conducted finally. The analyses were performed using SPSS (version 25, IBM Corp., USA), and a *p* value <0.05 was considered statistically significant.

## Results

A total of 421 pituitary adenoma patients who underwent EES were screened for inclusion in our study. 20 patients were excluded due to missing data and 1 patient was excluded for a serious complication (rupture of the left internal-carotid-artery during surgery). Finally, 400 patients were included for analysis. The clinical characteristics of patients are detailed in Table 1.

**Table 1:** Patient demographics.

Variable	No CSF Leak (*n* = 386)	CSF Leak (*n* = 14)	*p* Value
Age (years)	48.5 ± 12.9	49.3 ± 12.5	0.821
Gender (no.)			0.864
Male	202	7	
Female	184	7	
BMI (kg/m^2^)	25.7 ± 3.9	23.8 ± 1.9	0.058
Tumor size (mm)			
Anteroposterior diameter	23.0 ± 10.2	28.6 ± 15.8	0.204
Transverse diameter	22.8 ± 9.8	29.6 ± 12.9	0.071
Vertical diameter	24.0 ± 11.8	29.1 ± 12.9	0.119
Knosp grade (no.)			0.564
0	19	0	
I	109	2	
II	96	4	
III	76	5	
IV	86	3	
Suprasellar extension grade (no.)			0.015
0	32	0	
A	183	3	
B	95	3	
C	76	8	
First transsphenoidal surgery (no.)			0.524
Yes	316	10	
No	70	4	
Intraoperative CSF leak (no.)			0.009
Yes	99	8	
No	287	6	
Use of pedicled nasoseptal flap (no.)			0.119
Yes	53	4	
No	333	10	
Lumbar drain (no.)			0.000
Yes	34	7	
No	352	7	

There were 191 female patients and 209 male patients. The average patient age at surgery was 48.5 years (11–82 years). The average patient BMI was 25.6 kg/m^2^ (17.2–42.5 kg/m^2^). Among those patients, 257 (64.25%) were overweight or obese (BMI ≥ 24), 143 (35.75%) of healthy weight (BMI < 24). There were 326 (81.5%) patients underwent EES for the first time and 74 (18.5%) patients underwent EES again.

**Figure 4:**
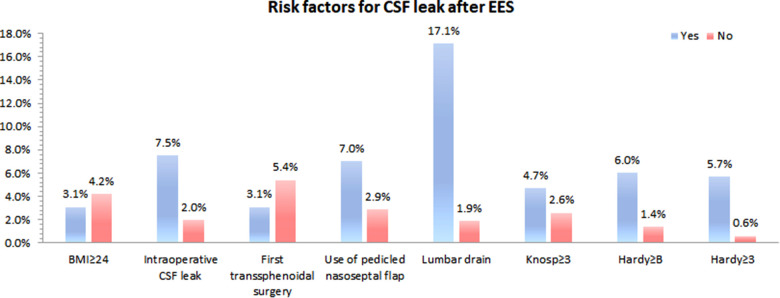
Effect of risk factors on rate of postoperative CSF leak.

Of the 400 patients enrolled for analysis, fourteen patients occurred postoperative CSF leak (3.5%). Figure 4 shows that the effect of risk factors on rate of postoperative CDF leak. There was no significant difference in age, gender, BMI, Knosp grade, repeated EES and use of pedicled nasoseptal flap between those with and without postoperative CSF leaks according univariate analysis (Table 1). Although there was no statistically significant difference, patients with postoperative CSF leakage had larger tumor sizes than those without postoperative leakage (Anteroposterior diameter 28.6 ± 15.8 mm vs. 23.0 ± 10.2 mm; *p* = 0.204) (Transverse diameter 29.6 ± 12.9 mm vs. 22.8 ± 9.8 mm; *p* = 0.071) (Vertical diameter 29.1 ± 12.9 mm vs. 24.0 ± 11.8 mm; *p* = 0.119).

There were 107 patients with CSF leakage during the operation. Patients with intraoperative CSF lake were more likely to develop postoperative CSF lake (with intraoperative CSF lake 7.5% [8/107] vs. without intraoperative CSF lake 2.0% [6/293]; *p* = 0.009).

Patients with higher grades of suprasellar extension (*p* = 0.015) and sellar floor erosion (*p* = 0.042) were more likely to develop postoperative CSF leak (Tables 1, 2). Patients with suprasellar extension grades B and C had a significantly higher leakage rate than those with less than B grades (6.0% [11/182] vs. 1.4% [3/218]; *p* = 0.024). Patients with sellar floor erosion grades III and IV had a significantly higher leakage rate than those with less than III grades (5.7% [9/159] vs. 0.6% [1/167]; *p* = 0.020). Patients with Knosp less than grade 3 appeared to have a lower rate of CSF leak, but this was not statistically significant (≥III 4.7% [8/170] vs. <III 2.6% [6/230]; *p* = 0.259) (Table 3).

**Table 2:** Sellar floor erosion grades of patients.

Variable	No CSF Leak (*n* = 316)	CSF Leak (*n* = 10)	*p* Value
Sellar floor erosion grade (no.)			0.042
I	10	0	
II	156	1	
III	81	5	
IV	69	4	

**Table 3:** Univariate analysis for predictors of postoperative CSF leak.

Variable	No CSF Leak	CSF Leak	*p* Value
BMI (kg/m^2^) (no.)	*n* = 386	*n* = 14	0.572
<24	137	6	
≥24	249	8	
Knosp grade (no.)	*n* = 386	*n* = 14	0.259
<III	224	6	
≥III	162	8	
Suprasellar extension grade (no.)	*n* = 386	*n* = 14	0.024
<B	215	3	
≥B	171	11	
Sellar floor erosion grade (no.)	*n* = 316	*n*= 10	0.020
<III	166	1	
≥III	150	9	

In 57 of 400 patients, pedicled nasoseptal flap was used for skull base reconstruction. Of these 57 patients, 4 (7.0%) patients developed postoperative CSF leakage. Leakage rates were relatively low in patients who did not use a nasoseptal flap (2.9% [10/343]). But there is no statistically significant difference (*p* = 0.119).

A total of 41 patients had a lumbar drain placed after operation (10.3%). Patients who underwent postoperative lumbar drain had a higher rate of CSF leakage compared with did not underwent postoperative lumbar drain (17.1% [7/41] vs. 1.9% [7/359]; *p* < 0.0001).

Multiple logistic regression analysis showed that intraoperative CSF leak and high grade of suprasellar extension were significantly associated with postoperative CSF leak (*p* < 0.05). Patients with intraoperative CSF leak were 3.75 times more likely to have a CSF lake when compared with those without intraoperative CSF leak. Patients with a suprasellar extension grade ≥ B were 4.29 times more likely to have a postoperative CSF leak when compared with those with a suprasellar extension grade < B (Table 4).

**Table 4:** Multivariate logistic regression for predictors of postoperative CSF leak.

Variable	*p* Value	OR (95% CI)
BMI ≥ 24 kg/m^2^	0.618	0.758 (0.255–2.254)
Not first transsphenoidal surgery	0.500	1.516 (0.452–5.080)
Intraoperative CSF leak	0.019	3.688 (1.238–10.987)
Suprasellar extension grade ≥ B	0.020	4.610 (1.266–16.786)
Anteroposterior diameter	0.688	1.013 (0.951–1.079)
Transverse diameter	0.202	1.040 (0.979–1.105)
Vertical diameter	0.445	0.975 (0.913–1.041)

## Discussion

EES is the preferred first-line treatment for pituitary adenomas as skull base tumors. CSF leak is one of the most common postoperative complications after EES for pituitary adenomas. According to literature reports, the incidence of CSF leak after EES for pituitary adenomas ranges from 2.6% to 12.1% (4, 11–17). In the current study, 3.5% of patients with pituitary adenomas developed cerebrospinal fluid leakage after EES. Similar to other studies (4, 13), there was no statistically significant difference in age, gender, and Knosp grade between those with and without postoperative CSF leaks in the present study.

It is reported that increased intracranial pressure in overweight and obese patients can place additional strain on skull base reconstruction, leading to increase the risk of postoperative CSF leak (4). It is proved that BMI can be a risk factor for postoperative CSF leak in EES for pituitary adenomas (5). But based on our data, there was no statistically significant difference in BMI between those with and without postoperative CSF leaks. The reason for this difference may be racial differences. The BMI of Asians is generally lower than that of Europeans and Americans. The difference in BMI may have little effect between the two groups of patients with and without CSF leak.

Consistent with reports in the literature (13, 18), our study showed that intraoperative CSF leak increases postoperative CSF leak rate. In this study, 107 (26.8%) patients developed intraoperative CSF leak. Eight patients with intraoperative CSF leak eventually developed postoperative CSF leak. In our experience, we perform rigorous skull base reconstruction and lumbar drainage in patients with intraoperative CSF leakage. Even so, these patients had a relatively high risk of postoperative CSF leak. Of these 8 patients with postoperative CSF leak, 5 patients underwent postoperative lumbar drainage, and 4 patients used nasoseptal flaps in skull base reconstruction. Intraoperative CSF leakage, especially high-flow leakage, can increase the difficulty of skull base reconstruction and increase the risk of postoperative CSF leak. Univariate and multivariate statistical analysis showed that intraoperative CSF leak can be used as a risk factor for postoperative CSF leak in this study.

Intraoperative CSF leak flow strongly affects postoperative CSF leak rate, as reported by Di Perna et al (19). High flow CSF leak (IHFL) was defined as large dural defect and basal cisterns or ventricular opening, while small dural defect and moderate CSF leak defined the low flow leak (ILFL) (20). In the study of Perna et al, Postoperative CSF leak rate, resulted higher in the IHFL group (25.5%) than in the ILFL group (10.5%) (19). In addition, the flow of intraoperative CSF leak determines different reconstruction strategies. Research has shown that, mucosal flap and inlay for high flow intraoperative CSF leak improved the postoperative CSF leak rate (21). Unfortunately, the intraoperative CSF leak flow was not recorded in our surgical records.

For the first time, we introduced the effect of suprasellar extension grades and sellar floor erosion grades on CSF leak after EES. The Hardy–Wilson classification was used for the assessment of suprasellar extension grades and sellar floor erosion grades (Figures 2, 3). Based on our data, suprasellar extension grades and sellar floor erosion grades can be used as risk factors for postoperative CSF leak after EES. Patients with higher grades of suprasellar extension and sellar floor erosion were more likely to develop postoperative CSF leak (Tables 1, 2). Patients with a higher grade of suprasellar invasion, especially those with tumor expansion into the ventricular system, have an increased risk of intraoperative CSF leakage, resulting in a relatively increased incidence of postoperative CSF leakage. Patients with a higher level of sellar floor erosion have an increased degree of dura destruction and increase difficulty in skull base reconstruction, thereby increasing the incidence of postoperative cerebrospinal fluid leakage.

With advances in the development of vascularized flaps in EES, pedicled nasoseptal flaps have been increasingly employed for skull base reconstruction. A systematic review found that vascularized flaps were associated with a lower rate of postoperative CSF leaks (22). Especially in the case of high-flow intraoperative CSF leakage, the pedicled vascularized flap has a more significant effect. In our study, although there was no statistically significant difference, use of a pedicled nasoseptal flap was associated with a higher rate of postoperative CSF leak. This association may be due to selection bias. As mentioned earlier, the vascularized flap plays an important role in reducing the incidence of postoperative CSF leakage. Currently, in our group, we routinely use pedicled nasoseptal flaps for skull base reconstruction in patients with a high risk of postoperative CSF leakage.

Lumbar drainage is often used in the perioperative period to reduce intracranial pressure and prevent postoperative CSF leaks following EES for skull base lesions (23, 24). A prospective, randomized controlled trial confirmed that perioperative lumbar drainage reduced the rate of postoperative CSF leaks after EES (6). Our study indicates that patients who underwent postoperative lumbar drain had a higher rate of CSF leakage compared with did not underwent postoperative lumbar drain. This situation is the same as the intraoperative pedicled nasoseptal flaps, which is caused by selection bias. In our study, lumbar drainage was typically placed in patients with high risk of postoperative CSF leakage. Therefore, the results of statistical analysis showed that patients with lumbar drainage were more prone to CSF leakage.

**Figure 5:**
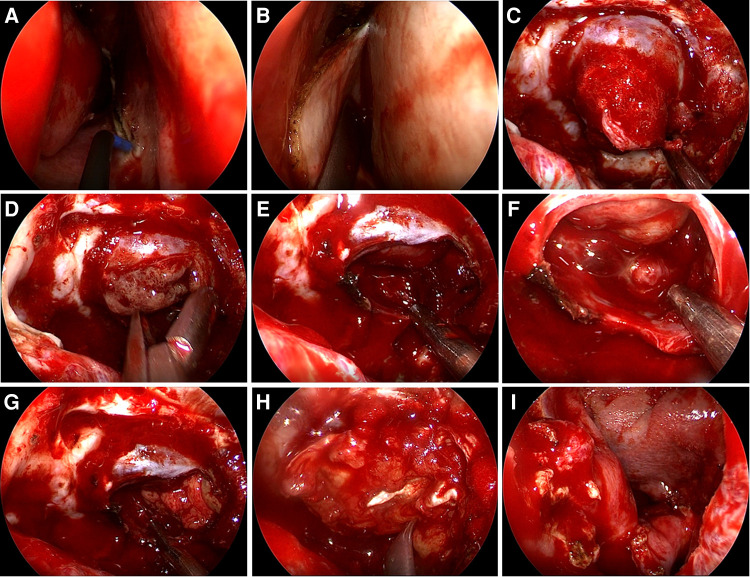
Rigorous skull base reconstruction procedure. (**A,B**) A pedicled nasoseptal flap was prepared in advance. (**C**) The tumor eroded the dura mater and protruded into the sphenoid sinus. (**D**) The tumor is removed in steps. (**E,F**) Pituitary adenoma has been completely removed and intraoperative CSF leakage occurred. (**G**) The fat graft was placed. (**H**) The fascia lata graft was placed. (**I**) The pedicled nasoseptal flap was placed over the fascia lata graft.

Free tissue grafts, vascularized flaps, gasket sealing and lumbar drains are most commonly used to prevent postoperative CSF leaks (25). Based on our data and the surgical experience of our team, for patients with high risk of postoperative CSF leakage, rigorous skull base reconstruction and perioperative lumbar drainage are beneficial to reduce the occurrence of postoperative CSF leakage. Rigorous skull base reconstruction includes various combinations of biomaterials, free tissue grafts (fat grafts and fascia lata grafts) and vascularized regional flaps. Figure 5 shows a rigorous skull base reconstruction procedure in a patient with high-risk postoperative CSF leak.

## Conclusions

Higher suprasellar extension grade, higher sellar floor erosion grade and intraoperative CSF leak were risk factors for postoperative CSF leak after endoscopic treatment of pituitary adenoma. Rigorous skull base reconstruction including use of a pedicled nasoseptal flap and perioperative lumbar drainage may avoid postoperative CSF leak.

## Limitation

This study is a retrospective study, and there is obvious selection bias in the two risk factors of nasoseptal flap and lumbar drainage. These two risk factors require further prospective studies to clarify their impact on postoperative CSF leakage. In addition, to avoid the impact of surgeons' experience on the study due to years of operation, we only counted cases for one year. Therefore, the number of cases is relatively small. Finally, factors such as reconstruction technique, intraoperative CSF leak flow, and history of previous radiation treatment were not accounted for in our analysis.

## Data Availability

The original contributions presented in the study are included in the article/Supplementary Material, further inquiries can be directed to the corresponding author/s.

## References

[1] Moreno CS Evans CO Zhan XQ Okor M Desiderio DM Oyesiku NM. Novel molecular signaling and classification of human clinically nonfunctional pituitary adenomas identified by gene expression profiling and proteomic analyses. Cancer Res. (2005) 65(22):10214–22. 10.1158/0008-5472.CAN-05-0884 16288009

[2] Loyo-Varela M Herrada-Pineda T Revilla-Pacheco F Manrique-Guzman S. Pituitary tumor surgery: review of 3004 cases. World Neurosurg. (2013) 79(2):331–6. 10.1016/j.wneu.2012.06.024 22732515

[3] Parikh A Adapa A Sullivan SE McKean EL. Predictive factors, 30-day clinical outcomes, and costs associated with cerebrospinal fluid leak in pituitary adenoma resection. J Neurol Surg Part B. (2020) 81(1):43–55. 10.1055/s-0039-1679896 PMC6997010 32021749

[4] Fraser S Gardner PA Koutourousiou M Kubik M Fernandez-Miranda JC Snyderman CH Risk factors associated with postoperative cerebrospinal fluid leak after endoscopic endonasal skull base surgery. J Neurosurg. (2018) 128(4):1066–71. 10.3171/2016.12.JNS1694 28598276

[5] Dlouhy BJ Madhavan K Clinger JD Reddy A Dawson JD O'Brien EK Elevated body mass index and risk of postoperative CSF leak following transsphenoidal surgery. J Neurosurg. (2012) 116(6):1311–7. 10.3171/2012.2.JNS111837 22443502 PMC3449304

[6] Zwagerman NT Wang EW Shin SS Chang YF Fernandez-Miranda JC Snyderman CH Does lumbar drainage reduce postoperative cerebrospinal fluid leak after endoscopic endonasal skull base surgery? A prospective, randomized controlled trial. J Neurosurg. (2019) 131(4):1172–8. 10.3171/2018.4.JNS172447 30485224

[7] Hadad G Bassagasteguy L Carrau RL Mataza JC Kassam A Snyderman CH A novel reconstructive technique after endoscopic expanded endonasal approaches: vascular pedicle nasoseptal flap. Laryngoscope. (2006) 116(10):1882–6. 10.1097/01.mlg.0000234933.37779.e4 17003708

[8] Moon JH Kim EH Kim SH. Various modifications of a vascularized nasoseptal flap for repair of extensive skull base dural defects. J Neurosurg. (2020) 132(2):371–9. 10.3171/2018.10.JNS181556 30738381

[9] Knosp E Steiner E Kitz K Matula C. Pituitary adenomas with invasion of the cavernous sinus space: a magnetic resonance imaging classification compared with surgical findings. Neurosurgery. (1993) 33(4):610–7. discussion 7-8. 10.1227/00006123-199310000-00008 8232800

[10] Araujo-Castro M Acitores Cancela A Vior C Pascual-Corrales E Rodriguez Berrocal V. Radiological knosp, revised-knosp, and Hardy-Wilson classifications for the prediction of surgical outcomes in the endoscopic endonasal surgery of pituitary adenomas: study of 228 cases. Front Oncol. (2021) 11:807040. 10.3389/fonc.2021.807040 35127519 PMC8810816

[11] Parasher AK Lerner DK Glicksman JT Miranda SP Dimentberg R Ebesutani D Drivers of in-hospital costs following endoscopic transphenoidal pituitary surgery. Laryngoscope. (2021) 131(4):760–4. 10.1002/lary.29041 32830866

[12] Taghvaei M Fallah S Sadaghiani S Sadrhosseini SM Tabari A Fathi M Surgical complications of endoscopic approach to skull base: analysis of 584 consecutive patients. Eur Arch Otorhinolaryngol. (2022) 279(6):3189–99. 10.1007/s00405-022-07256-3 35102476

[13] Wang M Cai Y Jiang Y Peng Y. Risk factors impacting intra- and postoperative cerebrospinal fluid rhinorrhea on the endoscopic treatment of pituitary adenomas: a retrospective study of 250 patients. Medicine. (2021) 100(49):e27781. 10.1097/MD.0000000000027781 34889229 PMC8663863

[14] Song S Wang L Qi Q Wang H Feng L. Endoscopic vs. microscopic transsphenoidal surgery outcomes in 514 nonfunctioning pituitary adenoma cases. Neurosurg Rev. (2022) 45(3):2375–83. 10.1007/s10143-022-01732-4 35230574

[15] Zhang J Wang Y Xu X Gu Y Huang F Zhang M. Postoperative complications and quality of life in patients with pituitary adenoma. Gland Surg. (2020) 9(5):1521–9. 10.21037/gs-20-690 33224827 PMC7667121

[16] Huang X Zhang X Zhou J Li G Zheng G Peng L Analysis of risk factors and preventive strategies for intracranial infection after neuroendoscopic transnasal pituitary adenoma resection. BMC Neurosci. (2022) 23(1):1. 10.1186/s12868-021-00688-3 34979913 PMC8725403

[17] Chibbaro S Signorelli F Milani D Cebula H Scibilia A Bozzi MT Primary endoscopic endonasal management of giant pituitary adenomas: outcome and pitfalls from a large prospective multicenter experience. Cancers (Basel). (2021) 13(14):3603. 10.3390/cancers13143603 34298816 PMC8304085

[18] Magro E Graillon T Lassave J Castinetti F Boissonneau S Tabouret E Complications related to the endoscopic endonasal transsphenoidal approach for nonfunctioning pituitary macroadenomas in 300 consecutive patients. World Neurosurg. (2016) 89:442–53. 10.1016/j.wneu.2016.02.059 26902781

[19] Di Perna G Penner F Cofano F De Marco R Baldassarre BM Portonero I Skull base reconstruction: a question of flow? A critical analysis of 521 endoscopic endonasal surgeries. PLoS One. (2021) 16(3):e0245119. 10.1371/journal.pone.0245119 33720937 PMC7959384

[20] Conger A Zhao F Wang X Eisenberg A Griffiths C Esposito F Evolution of the graded repair of CSF leaks and skull base defects in endonasal endoscopic tumor surgery: trends in repair failure and meningitis rates in 509 patients. J Neurosurg. (2018) 130(3):861–75. 10.3171/2017.11.JNS172141 29749920

[21] Cai X Yang J Zhu J Tang C Cong Z Liu Y Reconstruction strategies for intraoperative CSF leak in endoscopic endonasal skull base surgery: systematic review and meta-analysis. Br J Neurosurg. (2021):1–11. 10.1080/02688697.2020.1849548 33475004

[22] Soudry E Turner JH Nayak JV Hwang PH. Endoscopic reconstruction of surgically created skull base defects: a systematic review. Otolaryngol Head Neck Surg. (2014) 150(5):730–8. 10.1177/0194599814520685 24493791

[23] Cohen S Jones SH Dhandapani S Negm HM Anand VK Schwartz TH. Lumbar drains decrease the risk of postoperative cerebrospinal fluid leak following endonasal endoscopic surgery for suprasellar meningiomas in patients with high body mass Index. Oper Neurosurg (Hagerstown). (2018) 14(1):66–71. 10.1093/ons/opx070 29253284

[24] Allen KP Isaacson B Purcell P Kutz JW Jr. Roland PS. Lumbar subarachnoid drainage in cerebrospinal fluid leaks after lateral skull base surgery. Otol Neurotol. (2011) 32(9):1522–4. 10.1097/MAO.0b013e318232e387 21956598

[25] Khan DZ Ali AMS Koh CH Dorward NL Grieve J Layard Horsfall H Skull base repair following endonasal pituitary and skull base tumour resection: a systematic review. Pituitary. (2021) 24(5):698–713. 10.1007/s11102-021-01145-4 33973152 PMC8416859

